# Vibrational spectroscopy at electrolyte/electrode interfaces with graphene gratings

**DOI:** 10.1038/ncomms8593

**Published:** 2015-06-30

**Authors:** Ya-Qing Bie, Jason Horng, Zhiwen Shi, Long Ju, Qin Zhou, Alex Zettl, Dapeng Yu, Feng Wang

**Affiliations:** 1 Department of Physics, University of California, Berkeley, Berkeley, California 94720, USA; 2 State Key Laboratory for Mesoscopic Physics, Department of Physics, Peking University, Beijing 100871, China; 3 Kavli Energy NanoSciences Institute, University of California, Berkeley and Lawrence Berkeley National Laboratory, Berkeley, California 94720, USA; 4 Materials Sciences Division, Lawrence Berkeley National Laboratory, Berkeley, California 94720, USA

## Abstract

Microscopic understanding of physical and electrochemical processes at electrolyte/electrode interfaces is critical for applications ranging from batteries, fuel cells to electrocatalysis. However, probing such buried interfacial processes is experimentally challenging. Infrared spectroscopy is sensitive to molecule vibrational signatures, yet to approach the interface three stringent requirements have to be met: interface specificity, sub-monolayer molecular detection sensitivity, and electrochemically stable and infrared transparent electrodes. Here we show that transparent graphene gratings electrode provide an attractive platform for vibrational spectroscopy at the electrolyte/electrode interfaces: infrared diffraction from graphene gratings offers enhanced detection sensitivity and interface specificity. We demonstrate the vibrational spectroscopy of methylene group of adsorbed sub-monolayer cetrimonium bromide molecules and reveal a reversible field-induced electrochemical deposition of cetrimonium bromide on the electrode controlled by the bias voltage. Such vibrational spectroscopy with graphene gratings is promising for real time and *in situ* monitoring of different chemical species at the electrolyte/electrode interfaces.

## Abstract (web summary)

Understanding of electrolyte-electrode interfaces is limited due to the lack of suitable probing techniques. Here, the authors present a vibrational spectroscopy based on graphene gratings, which enables sensitive and interface-specific detection of molecular vibrations at electrolyte-electrode interfaces.

## Graphical abstract

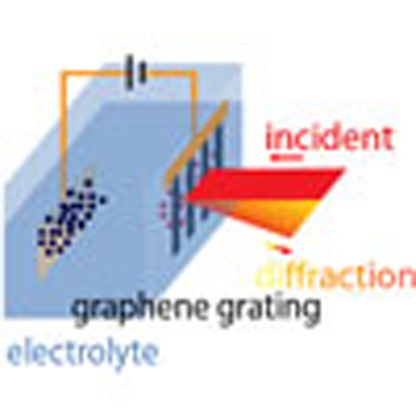

The electrolyte/electrode interface is of great interest for both fundamental science and applied technology: a rich variety of electrical field-dependent physical^1^,^2^,^3^ and chemical processes^4^,^5^ take place at this interface, which are important for applications such as supercapacitors^6^,^7^, batteries, fuel cells^7^,^8^ and electroplating^9^. However, microscopic understanding of the processes at electrolyte/electrode interfaces is quite limited due to lack of suitable experimental probing techniques^1^,^3^,^4^,^10^,^11^,^12^. For example, powerful surface techniques such as scanning tunnelling spectroscopy and many electron-based spectroscopy cannot access this interface. Optical spectroscopy can in principle probe buried interfaces and provide spectroscopic information of functional groups^13^,^14^,^15^, but its application in probing the electrolyte/electrode interface has to overcome several challenges: (1) both the electrolyte and conventional metal electrodes are opaque to infrared light^16^; (2) the interfacial signal can be easily overwhelmed by the bulk electrolyte signal; and (3) *in situ* detection with sub-monolayer sensitivity and surface specificity is difficult. In this paper, we report a new technique based on graphene gratings that overcomes these difficulties, which enables sensitive and interface-specific detection of molecular vibrations at the electrolyte/electrode interface. An infrared spectroscopy technique that overcomes all these difficulties will be a promising method for sensitive interface detection of molecules at the electrolyte/electrode interface.

Graphene is an attractive electrode for studies of electrolyte/electrode interfaces. It is stable and transparent to infrared light, and is being actively explored for applications in supercapacitors, batteries^17^, solar cells^18^ and displays^19^. In addition, the carrier concentration (or Fermi level) of graphene, a key electrode parameter for understanding the electric double layer and interfacial processes, can be directly determined through optical absorption spectroscopy^20^,^21^. A monolayer graphene grating further allows us to develop diffraction spectroscopy that greatly enhances the detection sensitivity of molecular vibrations and probes, specifically, molecules within the double layer at the electrolyte/electrode interface.

In this paper, we report a new vibrational spectroscopy technique based on diffraction of monolayer graphene gratings. This method overcomes the difficulties of probing buried interface with infrared, and enables sensitive and interface-specific detection of molecular vibrations spectra at the electrolyte/electrode interface. The advantages are demonstrated by detecting CH_2_ vibration peaks of polymer residue on graphene surface within seconds and the new spectroscopy enhances the relative contrast by ∼50 times compared with conventional absorption spectroscopy. Using the surfactant cetrimonium bromide (CTAB) as a model system, we observe CTAB adsorption on graphene and the reversible deposition/dissolution of CTAB at the graphene electrode with new diffraction vibrational spectroscopy.

## Results

### Diffraction spectroscopy design with graphene grating

**Figure 1:**
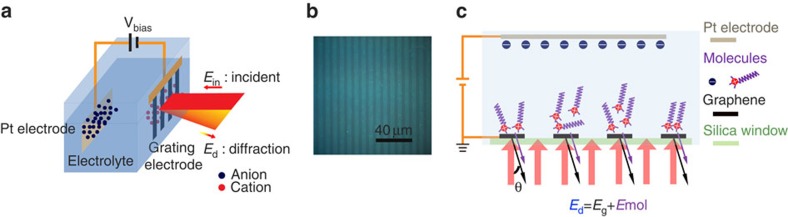
Schematic illustration of the spectroscopy design with graphene grating. (**a**) The electrochemical cell and spectroscopy configuration. Fused silica substrates are used as cell windows. The diffraction singals originate from an effective grating formed by periodic graphene/electrolyte and fused silica/electrolyte interfaces. The incident light electric field *E*_in_ is p-polarized, and the first-order diffraction is collected at the diffraction angle *θ*=25.9°. (**b**) Optical microscopic image of a graphene grating on fused silica. The bright ribbons are graphene and the grating period is 8 μm. (**c**) The diffraction signal is generated by a periodic variation of the optical susceptibility at the interface, which comes from both the graphene grating itself and the different adsorbed molecules like CTAB and chemical species in the electrolyte double layer induced by the graphene grating. Pt, platinum.

The novel ‘diffraction spectroscopy' based on graphene grating electrodes is designed to achieve high detection sensitivity and interfacial specificity study. The experimental scheme is illustrated in Fig. 1a. A femtosecond optical parametric amplifier is used as the light source, which produces tunable broadband infrared radiation with a bandwidth of ∼80 cm^−1^ and a repetition rate of 150 KHz. The infrared radiation is incident on a graphene grating electrode, which forms an electrochemical cell together with a platinum counter electrode and the aqueous electrolyte (Fig. 1a). To probe molecular species at the electrolyte/graphene electrode interface, we measure the first-order diffraction from the graphene grating rather than the transmission or reflection signal. Figure 1b shows the optical microscopy image of a typical graphene grating with 8 μm period on a fused silica substrate^22^. The diffraction signal originates from the periodic variation of optical susceptibility at the interface, which comes from not only the graphene grating itself but also the different adsorbed molecules and chemical species in the electrolyte double layer induced by the graphene electrode relative to that of the fused silica substrate (Fig. 1c). The bulk electrolyte solution beyond the nanometre-thick double layer region is homogeneous and contributes no diffraction signal. Therefore, this diffraction scheme provides excellent interface specificity.

In addition, the ‘diffraction spectroscopy' greatly enhances the detection sensitivity of the electrode-induced changes of molecular concentration at the interface. In conventional infrared absorption spectroscopy, the transmitted light intensity *I*_t_ results from interference between the incident light and the forward scattering of molecular species, that is, *I*_t_=|*E*_in_+*E*_mol_|^2^≈|*E*_in_|^2^+2·Re(*E*_in_*·*E*_mol_), where *E*_in_ and *E*_mol_ are the electrical field of incident light and molecular radiation, respectively, and |*E*_mol_|^2^ is omitted because |*E*_mol_|≪|*E*_in_|. The relative intensity change due to light absorption is therefore determined by Δ*I*_t_/*I*_t_≈2·Re(*E*_in_*·*E*_mol_)/|*E*_in_|^2^. For a monolayer of material, |*E*_mol_| from molecular vibration is typically <10^−3^ of *E*_in_ (ref. 23). So the absorption signal is usually ∼10^−3^ of the background light, and requires special infrared spectrometer and long-time average to obtain an absorption spectrum. In contrast, the diffracted signal is due to the interference between the diffraction of graphene electrode (*E*_g_) and the periodic modulation of molecular species within the electrical double layer (*E*_mol_) (Fig. 1c). Therefore, the relative intensity change in the diffraction signal is Δ*I*_d_/*I*_d_≈2·Re(*E*_g_*·*E*_mol_)/|*E*_g_|^2^. Because the diffracted electrical field from the graphene grating (*E*_g_) is about 1% of the incident electrical field *E*_in_ (refs 24, 25), the relative contrast of the molecular vibration signal in diffraction spectra will be enhanced by almost two orders of magnitude compared with direct absorption spectroscopy. In principle, this technique can be extended to ultrathin film metal gratings. However, it is practically difficult to form sub-nanometre or 1 nm thin continuous metal films. For thicker films, the diffraction from metal will lead to a strong background and diminish the advantage of the diffraction spectroscopy.

### Methylene stretching modes in diffraction spectroscopy

We demonstrate the capability of this vibrational spectroscopy based on graphene gratings by investigating CH_2_ stretching modes (within the spectral range of 2,800–3,000 cm^−1^) at the electrolyte/electrode interfaces in an electrochemical cell. The spectra of the diffracted infrared light from the graphene grating are taken with a two-dimensional InSb infrared camera.

**Figure 2:**
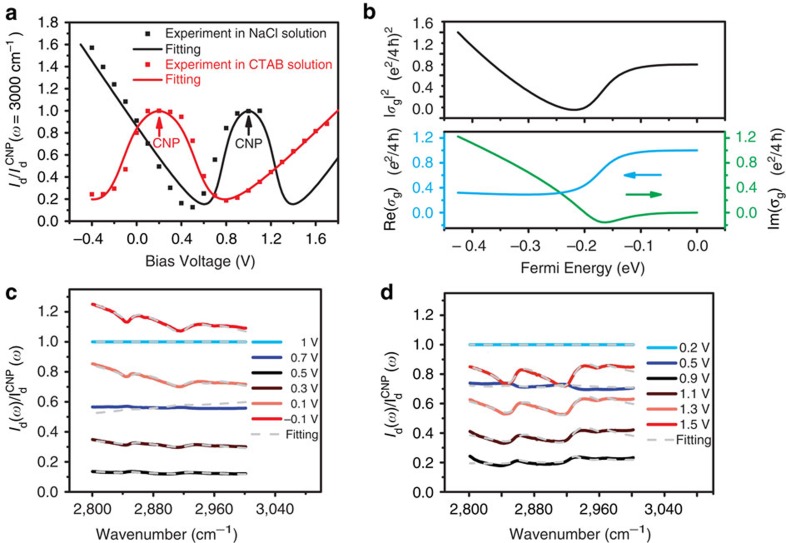
Bias-dependent diffraction spectra with graphene gratings in different electrolytes. (**a**) Normalized diffraction intensity *I*_d_/*I*_d_^CNP^ as a function of *V*_bias_ at 3,000 cm^−1^, which probes only the graphene grating response. The black dots and black line are experimental data and fitting, respectively, for graphene gratings in a 12 mM NaCl solution. The diffraction intensity shows a local maximum at CNP (*V*_bias_=1.0 V). The red dots and red line are experimental data and fitting, respectively, for graphene gratings in a 11 mM CTAB solution. *I*_d_ also exhibits a local maximum at CNP, which is shifted to *V*_bias_=0.2 V. (**b**) Simulation of the graphene conductivity in the NaCl solution as a function of Fermi level. Red, Re(*σ*_g_); green, Im(*σ*_g_); black, |*σ*_g_|^2^∝*I*_d_. (**c**,**d**) The solid lines show normalized diffraction spectra *I*_d_(*ω*)/*I*_d_^CNP^ (*ω*) at different *V*_bias_. Two CH_2_ vibrational resonances at 2,916 and 2,848 cm^−1^ are observed from polymer residues for the as-prepared graphene grating in a NaCl electrolyte (**c**). More pronounced CH_2_ resonances are observed for graphene gratings in the 11 mM CTAB electrolyte due to CTAB adsorption at the interface in (**d**). The dashed lines are theoretical fitting of the diffraction spectra as described in the text.

First, we examine the diffraction signal *E*_g_ from the graphene grating electrode using 12 mM NaCl solution as the electrolyte. Figure 2a shows the diffraction intensity at 3,000 cm^−1^ as a function of the bias voltage from 1.1 to −0.4 V (black squares), which is normalized by the intensity value at the charge neutral point (CNP) of graphene. The diffraction intensity at 3,000 cm^−1^ contains contribution mainly from the graphene grating because this frequency is away from any molecular vibration resonances in this system. The bias voltage of the electrochemical cell acts as an electrical gate that controls carrier concentration in graphene. At *V*_bias_=0 V graphene is strongly p-doped in the NaCl solution, and it becomes charge neutral at *V*_bias_=1.0 V. We observe that the diffraction intensity shows a local maximum at the CNP, which decreases upon hole doping to ∼10% of the CNP signal at *V*_bias_=0.5 V. Afterwards, the diffraction signal starts to increase with further hole doping. This non-monotonically change in infrared diffraction from the graphene grating originates from the gate-dependent complex optical conductivity in graphene^20^,^21^,^26^, since the first-order diffraction intensity from the graphene grating *I*_g_ is proportional to (Re(*σ*_g_))^2^+(Im(*σ*_g_))^2^, where Re(*σ*_g_) and Im(*σ*_g_) are the real and imaginary parts of graphene conductivity *σ*_g_, respectively. Figure 2b displays the calculated Re(*σ*_g_), Im(*σ*_g_) and |*σ*_g_|^2^ at 3,000 cm^−1^ as a function of the Fermi energy *E*_F_. Near the CNP, Re(*σ*_g_) from interband transitions dominates the diffraction signal. Re(*σ*_g_) decreases significantly when 2|*E*_F_| equals the incident photon energy due to Pauli blocking, and accounts for the reduced diffraction intensity from carrier doping initially. However, at high doping levels Im(*σ*_g_) from intraband transitions starts to dominate, which ultimately leads to diffraction intensity increasing at high |*E*_F_|. The solid line in Fig. 2a shows the good agreement of this model with the experimental data and fitting details can be find in Supplementary Note 1 and Supplementary Table 1.

Next we show the infrared diffraction spectra (from 2,800 to 3,000 cm^−1^) from as-prepared graphene electrodes in the 12 mM NaCl solution at different bias voltages. Figure 2c shows the normalized diffraction spectra *I*_d_(*ω*)/*I*_d_^CNP^(*ω*), where *I*_d_^CNP^(*ω*), the spectrum at CNP, is used as the reference. We observed two sharp resonances at 2,916 and 2,848 cm^−1^ on top of a broad background signal. The broad background arises from the graphene electrode, which first decreases and then increases with higher hole doping controlled by *V*_bias_. The two resonances at 2,916 and 2,848 cm^−1^ correspond to the anti-symmetric and symmetric stretching vibrations of the methylene group (-CH_2_-), respectively. A weak resonance at 2,960 cm^−1^ corresponding to methyl group (-CH_3_) is also present. The appearance of CH_2_ vibration peaks indicates that there are hydrocarbons on top of as-prepared graphene in NaCl solution, presumably due to the residue from the polymer transfer and/or photolithography processes. The diffraction spectra in Fig. 2c result from the interference between the graphene and CH_2_ vibration signals, and their understanding requires quantitative description of diffracted electrical field from both graphene and CH_2_ vibrations at different bias voltages. The normalized diffraction intensity is described by


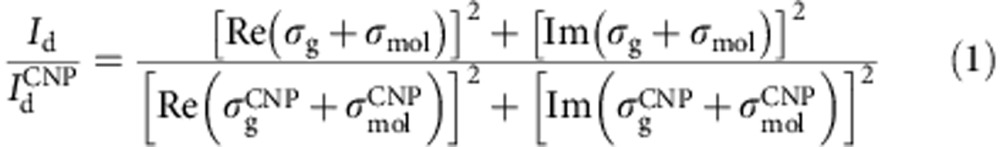


where *σ*_g_ and *σ*_mol_ are the effective complex conductivity for graphene and the adsorbed molecules, respectively. *σ*_g_^CNP^ and *σ*_mol_^CNP^ are corresponding values near CNP. The derivation of equation (1) and the graphene complex conductivity *σ*_g_ is described in Supplementary Note 1 and in Supplementary Fig. 1, and the molecular responses from CH_2_ vibrations *σ*_mol_ can be described by a Lorenz model (Supplementary Note 2). We find that the experimental data can be well fitted by a constant amount of CH_2_ functional groups radiation interfering with the gate-dependent graphene radiation at different carrier concentration. Dashed silver lines in Fig. 2c show the fitting result using equation (1). Here we have used an averaged CH_2_ concentration of 1.1 × 10^15^ cm^−2^, as well as an oscillator strength of 1.372 and 0.63, respectively, for the CH_2_ anti-symmetric and symmetric stretching modes^27^. The change of line shape of the vibration resonance at different voltages is due to the interference between the molecular and graphene responses. More fitting details are discussed in Supplementary Note 2 and in Supplementary Fig. 2.

We note that the CH_2_ concentration corresponds to on average one CH_2_ group per unit cell in graphene, that is, an ‘effective' monolayer coverage, although the actual distribution of hydrocarbon is likely to be randomly distributed with relatively large clusters. And this effective monolayer coverage leads to a change of diffraction intensity of ∼6% from the graphene grating (Fig. 2c). This is in contrast to only ∼10^−3^ change for monolayer CH_2_ absorption in conventional transmission measurements. Indeed, we observe the CH_2_ peaks from organic residue at 10^−3^ level in similarly prepared graphene samples using conventional Fourier transform infrared spectroscopy with hours of average. Using the diffraction spectroscopy with graphene gratings, the relative signal change is enhanced by ∼50 times, and it enables us to obtain monolayer CH_2_ absorption spectra within seconds.

### Observation of adsorbed CTAB in the diffraction spectra

The vibrational spectroscopy based on graphene grating, with its high sensitivity and interface specificity, enables us to *in situ* monitor the accumulating process of molecular species at the electrolyte/graphene electrode interface. Here we use a cationic surfactant CTAB (CH_3_(CH_2_)_15_N(CH_3_)_3_Br) as a test system. CTAB have been widely used to assist DNA extraction^28^, gold nanoparticle synthesis^29^ and carbon nanotube dispersion^30^.

We plot in Fig. 2a (red squares) the gate-dependent diffraction signal at 3,000 cm^−1^ from the graphene grating in 11 mM CTAB solution. Compared with graphene in NaCl solution, the voltage dependence shows a similar behaviour except that the CNP is strongly shifted to *V*_bias_=0.2 V. Apparently CTAB largely cancels the strong p-doping in as-prepared graphene, presumably due to the adsorption of cation CH_3_(CH_2_)_15_N(CH_3_)_3_^+^ ( abbreviated as CTA^+^) that induces a significant amount of electrons in graphene.

The diffraction spectra near CH_2_ vibration frequencies from the CTAB solution/graphene electrode interface at different bias voltages are shown in Fig. 2d. The CNP spectrum at *V*_bias_=0.2 V is used as the reference. The spectra are reproducible in many cycles when the bias voltage is varied between −1 and 1.5 V, and there is a finite hysteresis when scanning direction switches. The bias-dependent spectra are quite similar to that in NaCl solution (Fig. 2c) except that the area of CH_2_ peak is two times larger. It indicates a higher CTAB concentration near the graphene electrode than that near bare fused silica substrate. A quantitative fitting of the spectra (dashed lines in Fig. 2d) can be achieved by assuming a constant excess CTAB molecule surface density of 1.9 × 10^14^ cm^−2^, corresponding to effectively 0.16 CTAB molecule per unit cell of graphene. The adsorbed CTAB molecules are likely to be disordered and isotropic since the spectral weight ratio for symmetric CH_2_ and anti-symmetric CH_2_ modes resembles that of isotropic ensemble of CTAB molecules.

### Evolution of the diffraction spectra under large bias voltage

**Figure 3:**
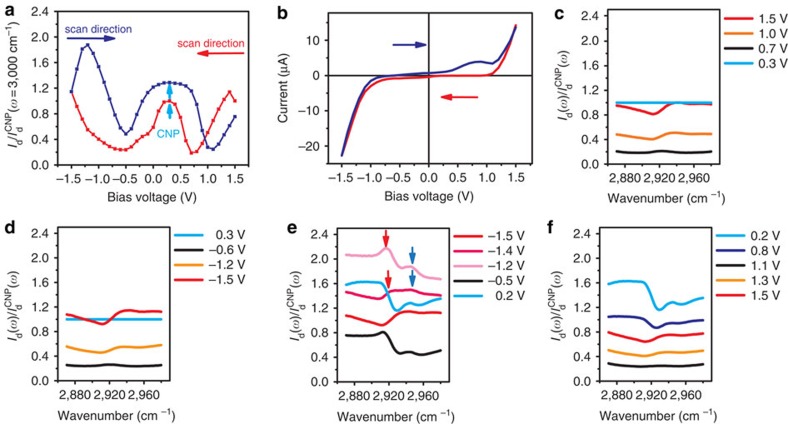
Electrochemical deposition/dissolution process monitored by diffraction spectroscopy. (**a**) Diffraction intensity *I*_d_ from the interface at 3,000 cm^−1^ as a function of the bias voltage *V*_bias_. *V*_bias_ was scanned from 1.5 to −1.5 V (red), and then back to 1.5 V (blue). (**b**) The cyclic voltammetry current shows a significant increase of current flowing through the interface at *V*_bias_=−1.5 V during the negative scan, and an extra peak at *V*_bias_=1 V during the positive scan. (**c**–**f**) Diffraction spectra from 2,870 to 2,980 cm^−1^ near the anti-symmetric CH_2_ vibration during the voltage scanning. All the spectra are normalized by the initial CNP spectrum at *V*_bias_=0.3 V. (**c**,**d**) Vibrational spectra with *V*_bias_ scanning from 1.5 to −1.5 V show behaviour similar to that in Fig. 2d. It can be described by a constant amount of CH_2_ group but a varying graphene dielectric constant. (**e**) Vibration spectra with *V*_bias_ scanning from −1.5 to 0.2 V. Total light diffraction intensity increases after *V*_bias_ has reached −1.5 V, the threshold for significant current increase in the cyclic voltammetry. At the same time, two new features near 2,920 cm^−1^ (red arrow) and 2,944 cm^−1^ (blue arrow) emerges. These spectra features indicate significant electrochemical deposition of crystalline CTAB after *V*_bias_ has passed −1.5 V. The electrochemically deposited CTAB molecules persists and leads to a resonance dip at the new CNP with *V*_bias_=0.2 V. (**f**) Vibration spectra with *V*_bias_ scaning from 0.2 to 1.5 V. The deposited CTAB are dissolved when *V*_bias_ is scanned over 1 V, and the spectral features become similar to those of the origninal state as in (**c**). This change in vibrational spectrum above *V*_bias_=1 V matches with the electrical current peak observed in the cyclic voltammetry.

To observe CTAB concentration change at the electrolyte/electrode interface due to electrochemical deposition and dissolution, we extend the negative voltage scan range. Specifically, we scanned the bias voltage from 1.5 to −1.5 V, and then back to 1.5 V in 1.4 mM CTAB solution. Figure 3a shows the bias-dependent diffraction intensity at 3,000 cm^−1^, and Fig. 3b is the corresponding cyclic voltammetry curve (see also Supplementary Fig. 3). Figure 3c–f display the diffraction spectra when the *V*_bias_ is scanned from 1.5 to −1.5 V (Fig. 3c,d), then from −1.5 to 1.5 V (Fig. 3e,f). Here we focus on the anti-symmetric CH_2_ vibrations in the spectral range of 2,870–2,980 cm^−1^ and all the spectra are normalized by the initial CNP spectrum at *V*_bias_=0.3 V.

When *V*_bias_ is varied between 1.5 and −1.2 V (Fig. 3c,d), the evolution of the spectra is similar to that in Fig. 2d, and it can be accounted for by the interference of graphene grating and a constant amount of CH_2_ groups at the interface. Spectra with electron or hole doping are largely symmetric because the graphene intrinsic response is symmetric. When *V*_bias_ reaches −1.5 V and starts to scan back, we see very different behaviour in the diffraction spectra as shown in Fig. 3e. First, we observe a strong increase in the overall diffraction intensity, indicating significant electrochemical deposition of materials at the graphene electrode. At the same time, we observe new spectral features emerging around 2,920 cm^−1^ (red arrow) and 2,944 cm^−1^ (blue arrows). The evolution of these spectra features as a function of electrical potential and their fittings are shown in Supplementary Note 3 and Supplementary Figure 4.

Previous studies of CTAB have shown that a new vibrational resonance around 2,943 cm^−1^ is associated with the head group of CH_3_-(N^+^) in crystalline CTAB^31^,^32^, and its presence indicates the formation of highly ordered CTAB at the graphene electrode. Therefore, our observed potential-dependent diffraction spectra changes indicate that once the *V*_bias_ reaches −1.5 V, a new electrochemical process is triggered to deposit highly ordered CTAB on the graphene electrode. This deposition process can be monitored in real time with our diffraction spectroscopy. When the bias voltage is further increased to positive, the deposited CTAB systematically modify the diffraction spectra. At the CNP with *V*_bias_=0.2 V, the normalized spectrum shows distinct features rather than a constant. When *V*_bias_ is further scanned beyond 1 V, however, the features associated with the deposited CTAB layer become weaker and then disappears, and the spectral shape becomes similar to those in Fig. 3c,d. It indicates that the deposited CTAB layers are being dissolved at these bias voltages. The observed deposition and dissolution of CTAB layers is also monitored with the cyclic voltammetry curve in Fig. 3b, where we observe a significant increase of current at *V*_bias_=−1.5 V in the negative scan, coincidence with the start of CTAB deposition, and a peak around *V*_bias_=1 V in the positive scan, coincidence with the disappearing of CTAB deposition. After the *V*_bias_ reaches 1.5 V, most of the deposited CTAB is dissolved, and the graphene electrode recovers to its initial status. When *V*_bias_ is scanned from 1.5 to −1.5 V afterwards, the diffraction spectra display the same behaviour as that in Fig. 3c (see in Supplementary Fig. 5).

The origin of the observed electrochemical deposition and dissolution of CTAB layers is not clear yet. When the graphene electrode is acting as an anode at *V*_bias_=−1.5 V, it can potentially oxide Br^−^ into Br_2_ at the electrode. In this case, one may expect the corresponding CTA^+^ cation reduction and deposition in the platinum counter electrode. However, we observe the deposition of CTAB layers at the graphene electrode. Further theoretical investigation will be required to understand microscopically the unconventional deposition processes taking place at the interfaces.

## Discussion

We demonstrated a vibration spectroscopy at the electrolyte/electrode interfaces using graphene grating electrodes. Its high detection sensitivity and interface specificity enables fast and *in situ* monitoring of molecular vibrations at the electrochemical interface. In this proof-of-principle study we used CH_2_ vibrations as an example, but the technique can be readily applied to other functional groups at different infrared frequency ranges. The diffraction spectroscopy enhances the relative contrast by ∼50 times compared with conventional absorption spectroscopy. Currently we demonstrate a sub-monolayer detection sensitivity, which is mainly limited by our laser fluctuation. With improved laser stability and/or balanced detection, we envision that the technique can probe as low as about 1% of a monolayer coverage and provides a powerful tool to investigate fundamental processes at the electrolyte/electrode interfaces in active electrochemical cells.

## Methods

### Sample preparation

In this experiment, graphene is grown on Cu foil by chemical vapour deposition method^33^ and then transferred to 0.5-mm thick fused silica substrates^33^. The periodical grating structure is patterned using standard photolithography and oxygen plasma etching^22^, and the graphene grating size is 7.5 × 7.5 mm. The electrolyte cell is constructed using Teflon and is thoroughly cleaned with acetone and ultrapure water before each measurement. All chemicals including NaCl and CTAB powder (99.99% purity) are obtained from Sigma-Aldrich.

### Infrared spectroscopy

The infrared radiation is generated by a femtosecond laser source. Specifically, an amplified femtosecond laser system (Pharos, Light Conversion Ltd) delivers laser pulses at 1,026 nm with a pulse duration of 260 fs and a repetition rate of 150 KHz. The laser amplifier pumps a broadly tunable optical parametric amplifier (Orpheus, Light Conversion Ltd) covering wavelengths from 600 to 2,200 nm. The mid-infrared wavelengths are generated by mixing the pump laser (1,026 nm) and the optical parametric amplifier output through difference frequency generation. The output infrared beam at around 3,000 cm^−1^ is collimated and sent to graphene at normal incidence. The first-order diffraction light is around 25.9° to the normal. The diffraction light is sent to a liquid nitrogen-cooled InSb camera with128 × 128 pixel arrays. The wavelength is then calibrated by absorption of thin film CTAB with its Fourier transform infrared spectrum. The detailed spectroscopy design is shown in Supplementary Fig. 6.

## 

## Additional information

**How to cite this article**: Bie, Y.Q. *et al.* Vibrational Spectroscopy at Electrolyte/Electrode Interfaces with Graphene Gratings. *Nat. Commun.* 6:7593 doi: 10.1038/ncomms8593 (2015).

## Supplementary Material

Supplementary Information Supplementary Figures 1-6, Supplementary Table 1, Supplementary Notes 1-3 and Supplementary References
