# Supplementary material for: Vibrational spectroscopy at electrolyte/electrode interfaces with graphene gratings
Source: Nat Commun. 2015 Jun 30;6:7593. doi: 10.1038/ncomms8593 (PMC4491813; doi:10.1038/ncomms8593)
Supplement: Supplementary Information — Supplementary Figures 1-6, Supplementary Table 1, Supplementary Notes 1-3 and Supplementary References [file ncomms8593-s1.pdf]

Supplementary Information for

Vibrational Spectroscopy at Electrolyte Electrode Interfaces with Graphene Gratings

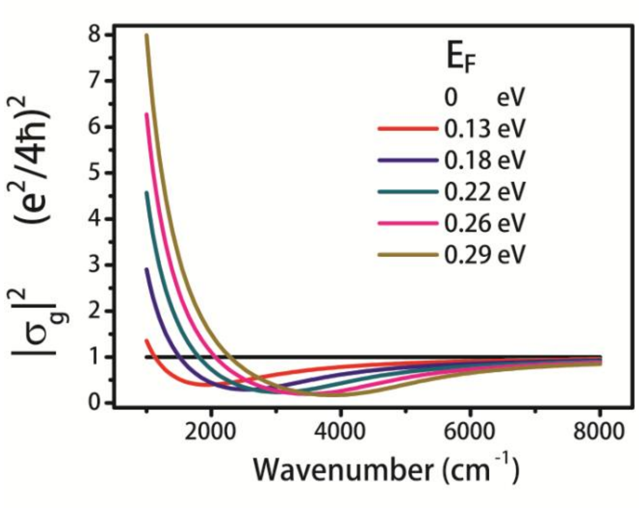

Supplementary Figure 1. Simulated $|\sigma_g|^2$ from pristine graphene gratings at different Fermi energy E_F. Intensity of diffraction spectra is proportional to $|\sigma_g|^2$.

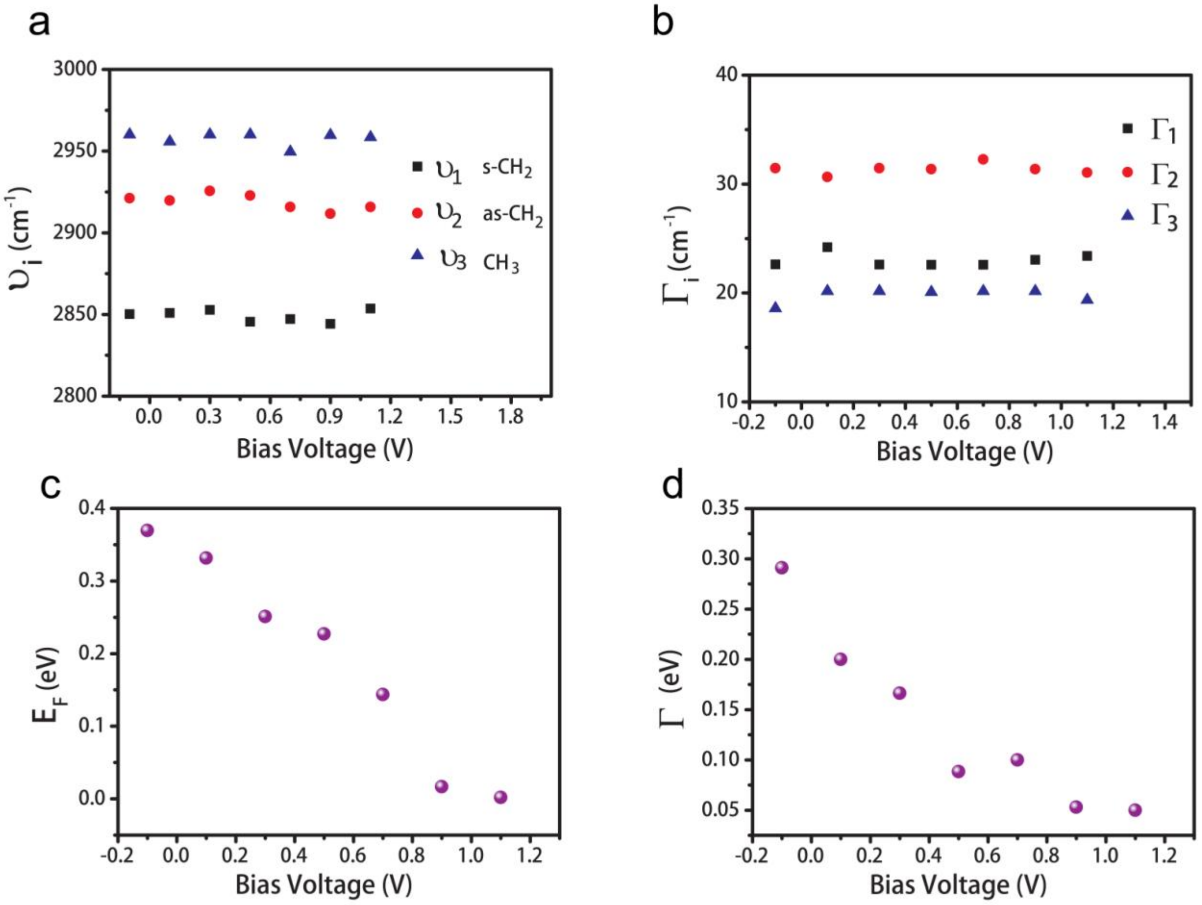

Supplementary Figure 2 Fitted parameters of interference between graphene grating diffraction and molecule resonance diffraction as bias voltage is increasing in 12 mM NaCl electrolyte. (a) molecular vibration resonance peak position; (b) molecular vibration full width half maximum; (c) graphene Fermi level; (d) graphene interband broadening. Charge neutral point voltage V_{CNP} = 1.0 V.

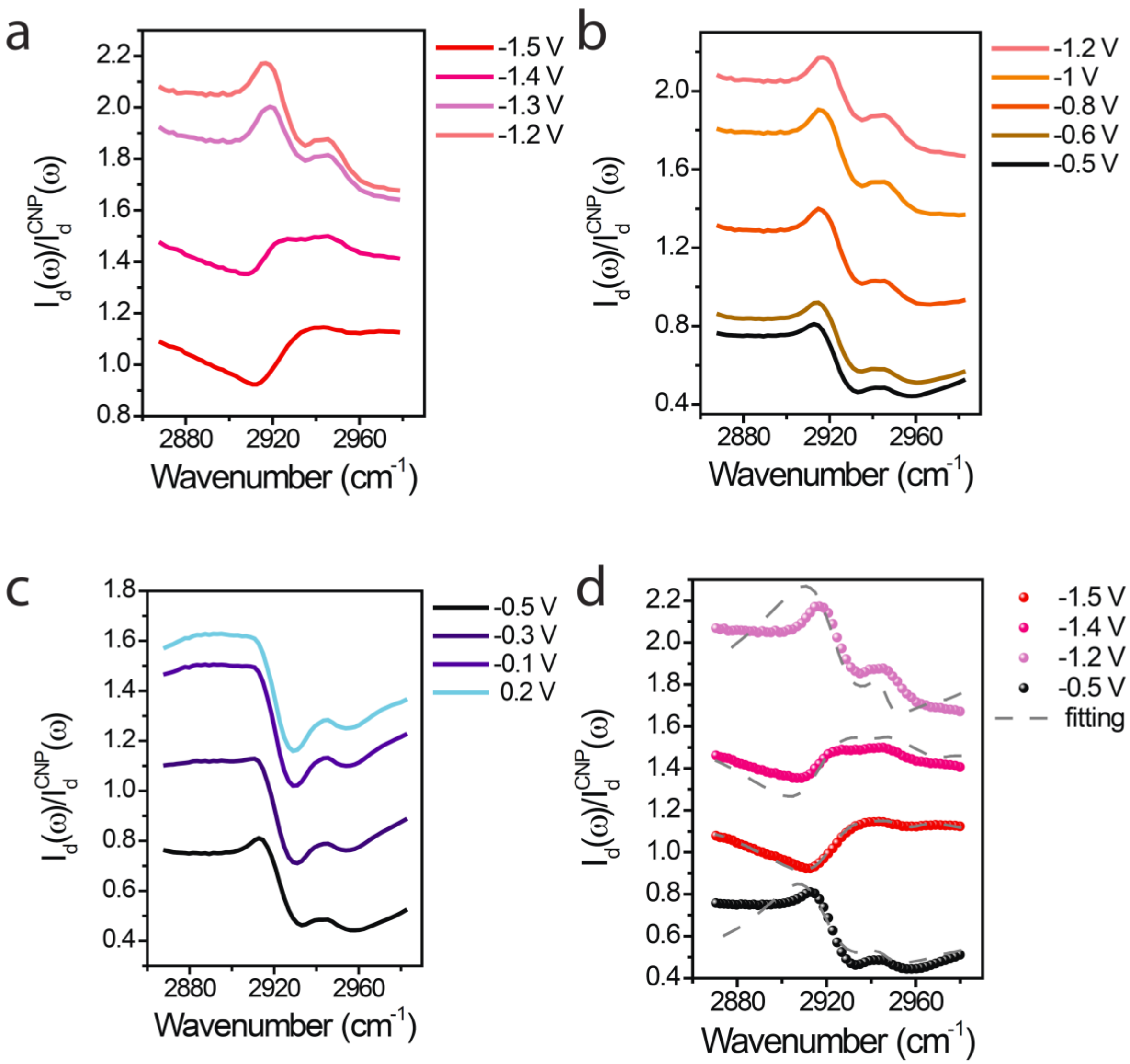

Supplementary Figure 3 Electrochemical deposition process monitored by diffraction spectroscopy. (a, b, c) More diffraction spectra with finer voltage scan corresponding to the process shown in Figure 3e are added to clearly show the evolution trend of the electrochemical reaction process. **(d)** Fitted spectra are included as the silver lines in addition to the experiment results using the CTAB deposition model.

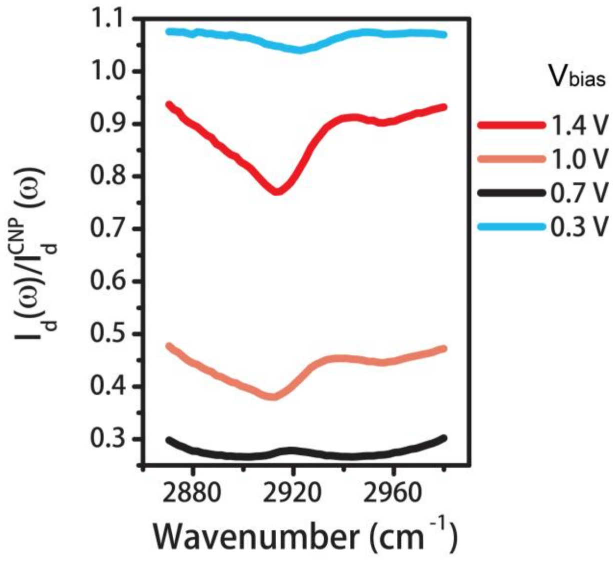

Supplementary Figure 4 Electrochemical dissolution process monitored by diffraction spectroscopy. The diffraction spectra display the similar behavior as that in Figure 2d and indicate that most of deposited CTAB layers are dissolved.

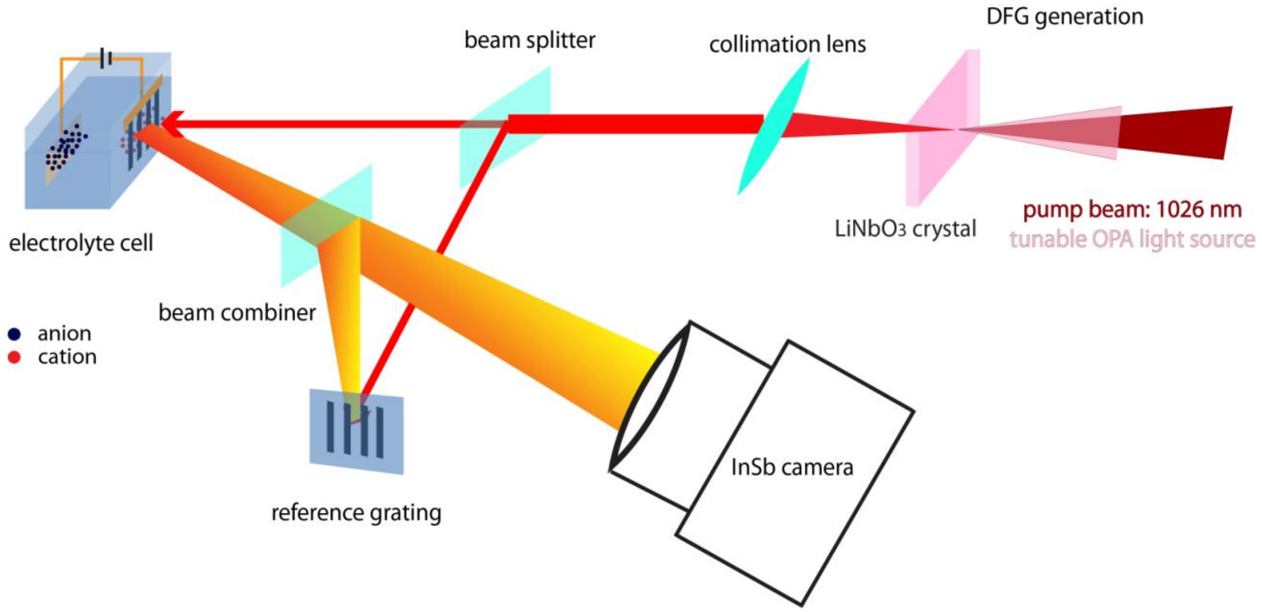

Supplementary Figure 5. **Sketch of our detailed spectroscopy design.** The infrared radiation is generated by a femtosecond laser source. Specifically, an amplified femtosecond laser system (Pharos, Light Conversion Ltd) delivers laser pulses at 1026 nm with a pulse duration of 260 fs and a repetition rate of 150 KHz. The laser amplifier pumps a broadly tunable optical parametric amplifier (Orpheus OPA, Light Conversion Ltd) covering wavelengths from 600 nm-2200 nm. The mid-infrared wavelengths are generated by mixing the pump laser (1026 nm) and the OPA output through difference frequency generation. Then the infrared beam is collimated and then separated by a beam splitter and divided into two path ways. One is for measuring the electrolytic cell and the other is shining on a grating with the same groves design as a reference. With the reference beam, we can minimize the laser fluctuation effect. Finally the spectra will be collected by a liquid nitrogen cooled InSb camera with128 $\times$128 pixel arrays.

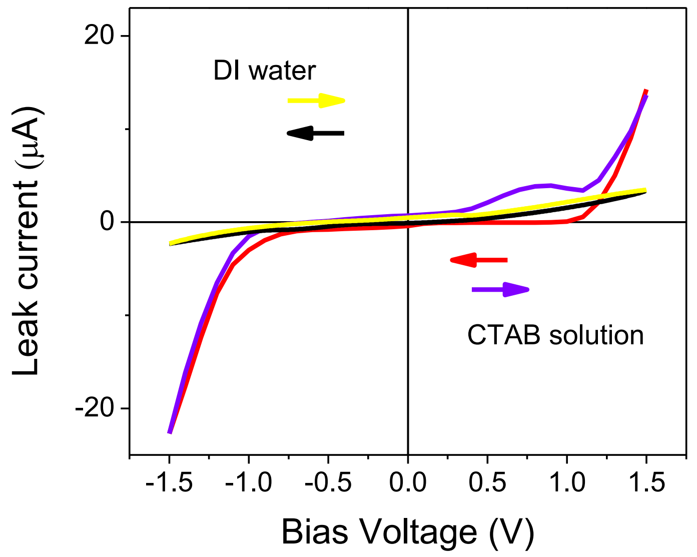

Supplementary Figure 6. **Comparison of cyclic voltammetry of CTAB solution and DI water.** With 1.4 mM CTAB solution, the cyclic voltammetry current shows a significant increase of current flowing through the interface at V_{bias} = -1.5 V during the negative scan, and an extra peak at V_{bias} = 1 V during the positive scan. In DI water, the leak current is significantly smaller and there is of no bump showing near 1V. This indicates the bump near 1V is related to CTAB.

Supplementary Table 1. Fitting parameters of graphene grating diffraction intensity at 3000 cm^{-1} for NaCl gating and CTAB gating

Fitting parameters	C ($/cm^2 \cdot V$)	A (eV/V)	Δ ($/cm^2$)
**NaCl gating**	7.3×10^{12}	0.26	2×10^{11}
**CTAB gating**	4.8×10^{12}	0.21	3.3×10^{11}

Supplementary Note 1:

Gate-dependent graphene diffraction response

From the scattering theory, the grating diffraction process can be treated as an incident light wave excites the graphene and the radiation from graphene grating eventually constructively interferes at the diffraction angle. The first order diffraction intensity from the graphene grating is described by

$$I_{\mathrm{g}} = \left|E_{\mathrm{g}}\right|^2 = \eta \cdot \left|\sigma_{\mathrm{g}} E_{\mathrm{in}}\right|^2 = \eta \cdot |E_{\mathrm{in}}|^2 \cdot [\left(Re(\sigma_{\mathrm{g}})\right)^2 + \left(Im(\sigma_{\mathrm{g}})\right)^2] \qquad \text{Eq. S (1)}$$

where E_{g} is the radiated electric field from graphene grating at the diffraction angle, η is a prefactor related to incident angle, polarization and grating geometry and σ_{g} is the complex conductivity of graphene. With molecules attached to graphene grating, the diffraction intensity is described by interference between the radiation from graphene and molecules. Therefore, the total diffraction intensity

$$I_{\mathrm{d}} = \left|E_{\mathrm{g}} + E_{\mathrm{mol}}\right|^2 = \eta \cdot |E_{\mathrm{in}}|^2 \cdot [\left(Re(\sigma_{\mathrm{g}}) + Re(\sigma_{\mathrm{mol}})\right)^2 + \left(Im(\sigma_{\mathrm{g}}) + Im(\sigma_{\mathrm{mol}})\right)^2 \qquad \text{Eq. S (2)}$$

where σ_{mol} is the complex conductivity of molecules. Equation (1) in the main text then can be derived straightforwardly.

σ_{g} ,the complex conductivity of graphene, contains contribution from both interband and intraband transitions in graphene, and its frequency dependence at different Fermi energies (E_{F}) can be approximated by [1,2]

$$Re(\sigma_{\mathrm{g}}) = \frac{e^2}{4\hbar}\left[1 + \frac{1}{\pi}\left(tan^{-1}\frac{E-2|E_{\mathrm{F}}|}{\Gamma} - tan^{-1}\frac{E+2|E_{\mathrm{F}}|}{\Gamma}\right)\right] + \frac{e^2}{\pi\hbar\tau}\frac{|E_{\mathrm{F}}|}{E^2+(1/\tau)^2} \qquad \text{Eq. S (3)}$$

$$Im(\sigma_{\mathrm{g}}) = \frac{e^2 E}{\pi\hbar}\frac{|E_{\mathrm{F}}|}{E^2+(1/\tau)^2} - \frac{e^2}{8\pi\hbar}ln\frac{(E+2|E_{\mathrm{F}}|)^2+\Gamma^2}{(E-2|E_{\mathrm{F}}|)^2+\Gamma^2} \qquad \text{Eq. S (4)}$$

where E is the incident photon energy, Γ the interband transition broadening. The free carrier scattering rate $1/\tau$ has little effect on the dielectric constant in our spectral range and can be approximated as zero. The Fermi level E_{F} varies with the carrier concentration n as $E_{\mathrm{F}} = h v_{\mathrm{F}}\sqrt{\pi n}$, where the Fermi velocity v_{F}

is set to 10^6 m/s. In the electrolytic cell, the averaged carrier concentration n_0 can be described by a capacitor model $n_0 = C(V_{\text{bias}} - V_{\text{CNP}})/e$, where C is fitted to be 1.17 and 0.77 $\mu F/cm^2$ for NaCl and CTAB gating, respectively. To account for the doping inhomogeneity in graphene, we introduced a local carrier concentration broadening of $\Delta = 2 \times 10^{11}/\text{cm}^2$. As a result, the conductivity of graphene can be described as

$$\sigma_{\text{g}}(n_0) = \frac{\int \sigma_{\text{g}}(n) e^{-(n-n_0)^2/\Delta^2} dn}{\int e^{-(n-n_0)^2/\Delta^2} dn} \qquad \text{Eq. S (5)}$$

The interband transition broadening Γ is assumed to be proportional to the carrier concentration and it's qualitatively described as $\Gamma = A|V_{\text{bias}} - V_{\text{CNP}}|$ in our simulation. The fitting results for the diffraction intensity as a function of V_{bias} are plotted in Fig. 2a (solid lines), where the fitting parameter are listed in Supplementary Table 1. Using the model described above, we can calculate the diffraction spectra from 1000 cm^{-1} to 8000 cm^{-1} for pristine graphene gratings at different E_F, as shown in Supplementary Fig. 1. No sharp resonance features are present for graphene response alone due to the broadband absorption of graphene.

Supplementary Note 2:

CH_2 vibrational resonances in the graphene-grating diffraction spectra

All experimental diffraction spectra in Fig.2c and 2d were fitted using Eq.(1), which includes optical responses of both graphene grating and periodic modulated molecular vibrations. Graphene response are described by Eq. S (1,3,4). For molecular part, the responses CH_2 and CH_3 vibrations can be described by the Lorentz model. Three vibrational resonances are present: the anti-symmetric CH_2, symmetric CH_2, and CH_3 resonances. The molecular optical susceptibility χ_{mol} and optical conductivity σ_{mol} are therefore characterized by

$$\chi_{\text{mol}} = \frac{e^2}{\varepsilon_0 m_{\text{CH2}} d_{\text{mol}} (2\pi c)^2}\left[\left(\frac{NA_1}{\nu_1^2-\nu^2-i\nu\cdot\Gamma_1}\right)+\left(\frac{NA_2}{\nu_2^2-\nu^2-i\nu\cdot\Gamma_2}\right)\right]+\frac{e^2}{\varepsilon_0 m_{\text{CH3}} d_{\text{mol}} (2\pi c)^2}\left(\frac{N'A_3}{\nu_3^2-\nu^2-i\nu\cdot\Gamma_3}\right) \quad \text{Eq. S(6)}$$

$$\sigma_{\text{mol}} = i\omega d_{\text{mol}} \varepsilon_0 \chi_{\text{mol}} \quad \text{Eq. S (7)}$$

Here N, N', m_{CH2}, m_{CH3}, d_{mol}, is, respectively, the CH_2, the CH_3 functional group density, CH_2 and CH_3 functional group mass, thin molecule film thickness. A_i (i = 1, 2, 3,) , ν_i (i = 1, 2, 3,) , and Γ_i (i = 1, 2, 3,) are oscillator strength, resonance wave number, and the broadening of each mode, which is symmetric CH_2, anti-symmetric CH_2 and CH_3 resonance in sequence. The fitting of Fig. 2c yields resonance peak positions and widths at around ν_1 = 2848 cm^{-1}, ν_2 = 2920 cm^{-1}, ν_3 = 2960 cm^{-1}, Γ_1 =25 cm^{-1}, Γ_2 =30 cm^{-1}, Γ_3 =20 cm^{-1} (Supplementary Fig.2 a, b), comparable to the established values. The fitting results for the graphene Fermi level E_F and the interband transition broadening Γ as a function of the bias voltage is shown in Supplementary Fig. 2c, d.

We can get the oscillator strengths A_1=0.63, A_2=1.372 from literature by assuming effective mass of CH_2 group as 14 proton mass[3]. Therefore we can estimate the density of CH_2 groups is ~1.1×10^{15} cm^{-2} on as prepared graphene gratings (Fig. 2c) and ~2.9×10^{15} cm^{-2} for graphene gratings in the 11 mM CTAB solution (Fig. 2d), which corresponding to 0.16 CTAB per unit cell of graphene.

Supplementary Note 3

The electrochemical deposition near graphene electrodes

Similar to the adsorption case, we modified the equation 1 and replaced optical conductivity of adsorbed molecule term σ_{mol} at high Fermi level with the optical conductivity of deposited CTAB term σ_{dep} and we get Eq. S(8)

$$\frac{I_{\mathrm{d}}}{I_{\mathrm{d}}{}^{CNP}} = \frac{[Re(\sigma_{\mathrm{g}}+\sigma_{\mathrm{dep}})]^2+[Im(\sigma_{\mathrm{g}}+\sigma_{\mathrm{dep}})]^2}{[Re(\sigma_{\mathrm{g}}{}^{CNP}+\sigma_{\mathrm{mol}}{}^{CNP})]^2+[Im(\sigma_{\mathrm{g}}{}^{CNP}+\sigma_{\mathrm{mol}}{}^{CNP})]^2} \qquad \text{Eq. S (8)}$$

$$\chi_{\mathrm{dep}}^{\mathrm{res}} = \frac{e^2 N_{\mathrm{dep}}}{\varepsilon_0 m_{\mathrm{CH2}} d_{\mathrm{dep}} (2\pi c)^2}\left[\left(\frac{A_4}{\nu_4^2-\nu^2-i\nu\cdot\Gamma_4}\right)+\left(\frac{A_5}{\nu_5^2-\nu^2-i\nu\cdot\Gamma_5}\right)+\left(\frac{A_6}{\nu_6^2-\nu^2-i\nu\cdot\Gamma_6}\right)\right] \qquad \text{Eq. S(9)}$$

$$\chi_{\mathrm{dep}}^{\mathrm{non-res}} = \alpha \qquad \text{Eq. S(10)}$$

$$\sigma_{\mathrm{dep}} = \sigma_{dep}^{res} + \sigma_{dep}^{non-res} = \mathrm{i}\omega d_{\mathrm{dep}}\varepsilon_0\left(\chi_{\mathrm{dep}}^{\mathrm{res}} + \chi_{\mathrm{dep}}^{\mathrm{non-res}}\right) \qquad \text{Eq. S (11)}$$

,where the σ_{dep} can be separated into two parts: $\sigma_{dep}^{non-res}$ the non-resonant contribution and σ_{dep}^{res} the resonant contribution of CTAB molecules. σ_{dep}^{res} is further described with the Lorentz model and is representing the resonances contribution in the spectrum range as described by Eq. S(9), Eq. S(11) , where χ_{dep} is the optical susceptibility of deposited CTAB molecules. In Eq. S(9), the most pronounced three resonance with ν_4 = 2850 cm^{-1}, ν_5 = 2918 cm^{-1}, ν_6 = 2944 cm^{-1}, Γ_4 =22 cm^{-1}, Γ_5 =30 cm^{-1}, Γ_6 =16 cm^{-1} are used in the fitting. The resonance at 2850 cm^{-1} and 2918 cm^{-1} are CH_2 symmetric and anti-symmetric stretching modes. The 2943 cm^{-1} has been assigned to symmetric stretching mode[4] for the head group of CH_3-(N^+). $\sigma_{dep}^{non-res}$ in Eq. S(10), is the non-resonance part of the optical conductivity of the deposited CTAB layer. It is related to the non-resonant susceptibility of CTAB layer $\chi_{\mathrm{dep}}^{\mathrm{non-res}}$ as shown in Eq. S(11). $\chi_{\mathrm{dep}}^{\mathrm{non-res}}$ is simply described by a real constant α =1.06 in our spectral range. The model is able to qualitatively reproduce the experimentally observed spectral features.

The deposition/dissolution process is reversible as shown in the Supplementary Figure 4, as the spectra for V_{bias} larger than 1V become similar to the spectra in Fig 2d where we have only adsorbed CTAB on graphene.

Supplementary References:

1 Mak, K. F. *et al.* Measurement of the optical conductivity of graphene. *Phys. Rev. Lett.* **101**, 196405 (2008).

2 Kim, J. *et al.* Electrical control of optical plasmon resonance with graphene. *Nano. Lett.* **12**, 5598-5602 (2012).

3 Milosevic, M. & Berets, S. L. Applications of the Theory of Optical Spectroscopy to Numerical Simulations. *Appl. Spectrosc.* **47**, 566-574 (1993).

4 Wang, W., Gu, B., Liang, L. & Hamilton, W. A. Adsorption and Structural Arrangement of Cetyltrimethylammonium Cations at the Silica Nanoparticle−Water Interface. *The Journal of Physical Chemistry B* **108**, 17477-17483, doi:10.1021/jp048325f (2004).
